# Inflammation markers and the risk of hypertension in people living with HIV

**DOI:** 10.3389/fimmu.2023.1133640

**Published:** 2023-03-21

**Authors:** Hui Ou-Yang, Hai-Yan Fu, Yu Luo, Zhao-Yuan Xu, Jun Liu, Rui Gao, Jin-Yu Duan, Ya-Chao Mao, Hong-Juan Li, Ying-Rong Du

**Affiliations:** ^1^ Department of Cardiovascular Medicine, The Third People’s Hospital of Kunming, Yunnan Clinical Medicine Center for Infectious Diseases, Kunming, China; ^2^ Department of Hospice Care, The Third People’s Hospital of Kunming, Yunnan Clinical Medicine Center for Infectious Diseases, Kunming, China; ^3^ Department of Infectious Diseases, The Third People’s Hospital of Kunming, Yunnan Clinical Medicine Center for Infectious Diseases, Kunming, China

**Keywords:** HIV, AIDS, hypertension, inflammation markers, high-sensitivity C-reactive protein

## Abstract

**Background:**

The incidence of hypertension is high in people living with HIV (PLWH). High-sensitivity C-reactive protein (hsCRP), systemic inflammation response index (SIRI), and neutrophil-to-monocyte ratio (NMR) are considered economic and convenient parameters that reflect the levels of inflammation in patients. Our aim was to explore whether indirect inflammation markers are associated with hypertension in PLWH.

**Methods:**

This was a case-control study. The case group (hypertension) comprised PLWH with hypertension, and the control group (non-hypertension) comprised sex- and age-(± 3 years)-matched PLWH without hypertension. Demographic parameters, hsCRP, neutrophil-to-lymphocyte ratio (NLR), platelet-to-lymphocyte ratio (PLR), systemic immune- inflammation index (SII), SIRI, lymphocyte-to-monocyte ratio (LMR), platelet-to-neutrophil ratio (PNR), platelet-to-monocyte ratio (PMR), NMR, time to HIV diagnosis, antiretroviral therapy (ART) duration, recent CD4^+^ and CD8^+^ cell counts, recent CD4^+^/CD8^+^ ratio, recent HIV viral load (HIV-RNA),and recent ART regimen were obtained from the patients’ electronic medical records. A t-test or Wilcoxon rank-sum test was performed to compare differences between the two groups, and conditional logistic regression was used to analyze the risk factors of hypertension. Correlations between inflammation markers and CD4^+^ cell counts, CD8^+^ cell counts, and CD4^+^/CD8^+^ ratio were analyzed using Spearman’s correlation.

**Results:**

In the hypertension group, body mass index (BMI), hsCRP, NLR, SII, SIRI, NMR, time to HIV diagnosis, ART duration, CD4^+^ and CD8^+^ cell counts, and CD4^+^/CD8^+^ ratio, the ratio of HIV-RNA < 100 copies/mL were all higher than those in the non-hypertension group, while the PNR was lower than that in the non-hypertension group. ART duration, CD4^+^ cell counts, HIV-RNA < 100 copies/mL, hsCRP, SIRI, and NMR were positively associated with hypertensive risk in PLWH. CD8^+^ cell counts and CD4^+^/CD8^+^ ratio was negatively associated with hypertensive risk in PLWH. SIRI was negatively correlated with CD4^+^ cell counts and CD8^+^ cell counts, but positively correlated with CD4^+^/CD8^+^ ratio.

**Conclusions:**

We identified positive associations between inflammation markers hsCRP, SIRI, NMR and hypertensive risk in PLWH. Alleviating inflammation may help control or delay the occurrence of hypertension in PLWH.

## 1 Introduction

With the increased use of antiretroviral therapy (ART), the incidence of opportunistic infections associated with human immunodeficiency virus (HIV) have significantly decreased, increasing the life expectancy of people living with HIV (PLWH) (1). However, the incidence of age-related hypertension in PLWH has increased. Although regular ART significantly lowers the level of inflammation in PLWH, it does not completely alleviate inflammation, and patients often maintain a persistent state of low-level inflammation (2, 3) independently of traditional cardiovascular risk factors, such as dyslipidemia, obesity, diabetes, and smoking (4). Chronic inflammation causes major alterations in physiological processes that play important roles in the pathogenesis and progression of cardiovascular diseases (5). PLWH are 2.16 times more likely to develop cardiovascular diseases than individuals without HIV. The proportion of PLWH with cardiovascular diseases has increased from 0.36% in 1990 to 0.92% in 2015 (6). Hypertension, the most common cardiovascular disease, is particularly prevalent in the PLWH population; approximately 1 in 4 PLWH has hypertension, and the prevalence increases with age (7). Therefore, there is an urgent need to explore the risk factors for hypertension in this population, and effective interventions should be implemented at early stages to delay the occurrence of hypertension. Many studies have shown that the risk factors for hypertension in PLWH include unhealthy lifestyle habits, high body mass index (BMI), hyperlipidemia, adverse effects from ART, CD4^+^ cell counts (8, 9), and CD4^+^/CD8^+^ ratio (10). A long-term micro-inflammatory environment may lead to hypertension in PLWH.

Certain parameters can indicate the body’s state of inflammation and affect the progression of some diseases. The neutrophil-to-lymphocyte ratio (NLR) and systemic immune-inflammation index (SII) can predict the occurrence and prognosis of cardiovascular events in non-HIV population (11–16). The NLR, combined with the platelet-to-lymphocyte ratio (PLR), can also predict the prognosis of acute myocardial infarction (17). Interestingly, NLR and PLR are independently associated with all-cause mortality in PLWH (18). However, the role of inflammation markers in predicting hypertension in PLWH remains unknown.

We retrospectively analyzed inflammation marker (high-sensitivity C-reactive protein (hsCRP) and eight inflammation parameters obtained during routine blood tests) levels and clinical factors in patients with and without hypertension who had also been diagnosed with HIV. We explored the associations between clinically accessible markers of inflammation and the risk of developing hypertension in PLWH.

## 2 Materials and methods

### 2.1 Study design and setting

This was a case-control study carried out in the Third People’s Hospital of Kunming, the largest PLWH-designated hospital in Kunming, China. The hospital provides HIV testing, treatment for HIV-related comorbidities, ART therapy, and follow-up services. The study was approved by the Ethics Committee of the Third People’s Hospital of Kunming (approval number: 2021120610). All information was collected by professionals and kept confidential.

### 2.2 Study population

**Figure 1:**
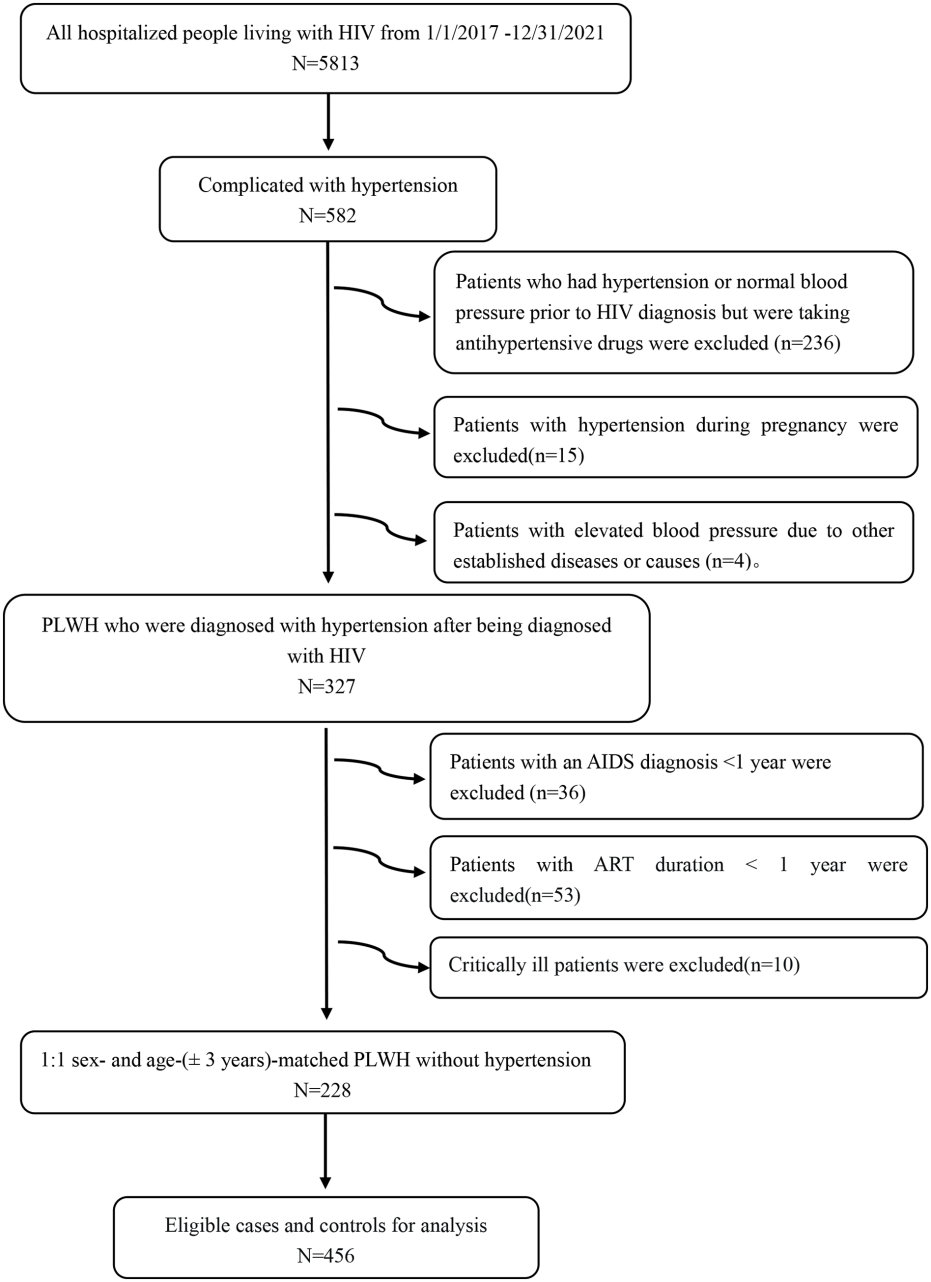
Study population flowchart. HIV, human immunodeficiency virus; AIDS, acquired immune deficiency syndrome; ART, antiretroviral therapy; PLWH, people living with HIV.

We recruited patients at least 18 years of age who were diagnosed with HIV and hospitalized in the Third People’s Hospital of Kunming from January 1, 2017 to December 31, 2021. The case group included PLWH who were diagnosed with hypertension after being diagnosed with HIV, and the control group included PLWH without hypertension. Patients in both groups were required to have had an acquired immune deficiency syndrome (AIDS) diagnosis for ≥1 year and to have underwent ART for ≥1 year. The electronic medical record (EMR) system search identified 582 patients with hypertension during the study period, after excluding patients who had hypertension or normal blood pressure prior to HIV diagnosis but were taking antihypertensive drugs (n=236), patients with hypertension during pregnancy (n=15), and patients with elevated blood pressure due to other established diseases or causes (n=4). After these exclusions, 327 patients who developed hypertension after being diagnosed with HIV were included. In accordance with the goal of the study, patients with an AIDS diagnosis <1 year (n=36), ART duration <1 year (n=53), or were critically ill (n=10) were also excluded. In the end, 228 PLWH were included in the case group (**Figure 1**). Similarly, in the study population, 228 PLWH without hypertension were randomly matched 1:1 by sex and age ( ± 3 years) with the control group. The diagnosis of hypertension was made by a consultation’s cardiovascular doctors according to the 2003 World Health Organization (WHO)/International Society of Hypertension (ISH) statement on management of hypertension (19) and the 2020 International Society of Hypertension Global Practice Guidelines for Hypertension (20). The diagnosis of HIV/AIDS was based on the Chinese HIV/AIDS Diagnosis and Treatment Guidelines from the year in which the patient was hospitalized (21–23).

### 2.3 Study variables

#### 2.3.1 Demographic parameters

The following research data were collected from the EMR system: age, sex, ethnicity, marital status, smoking, alcohol addiction status, family history of hypertension, BMI, length of HIV diagnosis, ART duration, and recent ART regimen. BMI was calculated as the patient’s weight in kilograms divided by the square of their height in meters, and height and weight measurements were taken when the patients were admitted (barefoot and in light clothing). Based on the criteria for Chinese adults, BMI was classified into the following four categories: underweight <18.5, normal 18.5–23.9, overweight 24–27.9, and obese ≥28.0 kg/m^2^ (24). However, considering PLWH generally have a relatively low weight compared to the general population, we classified underweight and normal as one category, namely BMI<24 kg/m^2^, and overweight and obese as another, defined as BMI ≥24 kg/m^2^. Smoking was defined as smoking ≥1 cigarette/day for at least 6 months consecutively or cumulatively. Alcohol addiction status was defined as drinking ≥80/g once a week for ≥1 year. The length of HIV diagnosis was the difference between the year of the initial HIV diagnosis by the patient as self-reported or laboratory-recorded and the year of admission, and ART duration was the difference between the year of initial ART treatment and the year of admission, as reported by the patient or recorded in the EMR system. ART drug was classified as: nucleoside reverse transcriptase inhibitors (NRTIs), nonnucleoside reverse transcriptase inhibitors (NNRTIs), protease inhibitors (PIs), and integrase strand transfer inhibitors (INSTIs).

#### 2.3.2 Laboratory indicators

On the first day of admission, blood samples were drawn by professionals from patients who had been fasting for at least 8 hours. Counts of the total neutrophil, monocyte, platelet, and lymphocyte were obtained using a hematology autoanalyzer (Sysmex, XG-550) and expressed as multiples of 10^9^/L. hsCRP was detected with an AU5400 automatic biochemical analyser.CD4^+^ cell counts, CD8^+^ cell counts, and CD4^+^/CD8^+^ ratio was determined using a flow cytometer (BD Company, New Jersey, USA), and the data were analyzed with Flow Jo software 2.8/3.0 (Tree star, Ashland, OR, USA). HIV primary screening tests were performed using an Enzyme-Linked Immunosorbent Assay (Taiwan Biotechnology, Beijing, China) according to manufacturer instructions, and HIV status was confirmed *via* western blot analysis. The plasma HIV viral load (HIV-RNA) was quantified by real-time RT- PCR Test (Roche, Mannheim, USA), an HIV-RNA level below 100 copies/mL was classified as undetectable. Calculations of the various parameters were performed as follows: NLR=neutrophil-to-lymphocyte ratio, PLR=platelet-to-lymphocyte ratio, SII=neutrophil/lymphocyte × platelet, systemic inflammation response index (SIRI)=neutrophil × monocyte/lymphocyte, LMR=lymphocyte-to-monocyte ratio, PNR=platelet-to-neutrophil ratio, PMR=platelet-to-monocyte ratio, and NMR=neutrophil-to-monocyte ratio. All test data were from the patients’ most recent hospital admission.

### 2.4 Statistical analyses

We defined the case group as the hypertensive group and the control group as the non-hypertensive group, conditional logistic regression was used to analyze risk factors for hypertension. Normally distributed measurement data are expressed as the mean ± standard deviation (SD), and non-normally distributed measurement data are presented as median (interquartile range, IQR). Enumeration data are expressed as constituent ratios. A t-test or Wilcoxon rank-sum test was used to compare the measurement data between the two groups, and a chi-squared test was used to compare the enumeration data. Conditional logistic regression analysis was performed on the factors with P<0.1 to analyze the risk factors of hypertension. Nine inflammation markers were treated as continuous variables, and categorical variables included ethnicity (1=Han, 2= minority), marital status(1= spinsterhood, 2=married, 3=divorce/widowhood), family history of hypertension(no=0,yes=1), smoking(no=0, yes=1), alcohol addiction status (no=0, yes=1), BMI (kg/m^2^)(< 24 = 1, ≥24 = 2), years of HIV diagnosis (1-4 = 1, 5-9 = 2, ≥10 = 3), years of ART duration (1-4 = 1, 5-9 = 2, ≥10 = 3), and number of ART classes (one=1, two=2, three=3), NRTIs (no=0, yes=1), NNRTIs (no=0, yes=1), PIs (no=0, yes=1), and INSTIs (no=0, yes=1). Correlations between hsCRP, SIRI, NMR, and HIV status (CD4^+^ and CD8^+^ cell counts and their ratio) were determined using Spearman’s correlation coefficients. All statistical analyses were performed using SPSS software version 20.0 (IBM Corporation, Armonk, New York, USA) and graphs were plotted using GraphPad Prism version 9 (GraphPad Software, Inc., San Diego, CA, USA). Statistical significance was defined as *P <*0.05.

## 3 Results

### 3.1 Participant characteristics

We accessed the medical records of 582 participants. Among them, 327 (56.2%) were PLWH diagnosed with hypertension after their HIV diagnosis. After patient exclusion based on the aforementioned criteria, medical records from 228 PLWH were included in the final analytical set (**Figure 1**). In addition, 228 PLWH without hypertension were enrolled as controls. Patients in both groups fulfilled the AIDS diagnosis for ≥1 year and ART duration for ≥1year criteria. The median age of the patients in the hypertensive and non-hypertensive groups combined was 51 years old. Patients were mainly between 40–49 and 50–59 years old.146 patients were male, accounting for 64.0% of the study participants. The Han nationality accounted for 90.8% of the hypertension group and 94.3% of the non-hypertension group, respectively. In the marital status, married accounted for 75.4% of the hypertension group and 69.3% of the non-hypertension group, respectively. A family history of hypertension was significantly more common in the hypertensive group than in the non-hypertensive group. Further, compared with the non-hypertensive group, the hypertensive group had a higher rate of smoking and alcohol addiction status. However, these differences were not statistically significant. Compared to the non-hypertensive group, the hypertensive group had a higher BMI value. The hypertensive group had a smaller proportion of underweight and normal weight patients, and a larger proportion of overweight patients. However, overall, underweight and normal weight (<24 kg/m^2^) patients accounted for the majority of patients in both groups. (**Table 1**). Regarding ART regimen, the patients in both groups were mainly treated with three-drug regimen, and the most frequent ART regimen was tenofovir disoproxil fumarate plus lamivudine plus efavirenz. Almost all participants received NRTIs, and only a minority received INSTIs (**Supplemental Table 1**).

**Table 1:** Demographic parameters of Patients in Both Groups.

Demographic parameters	HTN (n=228)	Non-HTN (n=228)	*P* Value
**Age (years)** ^a^	51 (45,58)	51 (45,58)	
18~39^b^	15 (6.6)	17 (7.5)	
40~49^b^	78 (34.2)	75 (32.9)	
50~59^b^	82 (36.0)	85 (37.3)	
≥60^b^	53 (23.2)	51 (22.4)	
**Sex (male)** ^b^	146 (64.0)	146 (64.0)	
**Ethnicity** ^b^			0.15
Han	207 (90.8)	215 (94.3)	
Minority	21 (9.2)	13 (5.7)	
**Marital status** ^b^			0.32
Spinsterhood	21 (9.2)	24 (10.5)	
Married	172 (75.4)	158 (69.3)	
Divorce/widowhood	35 (15.4)	46 (20.2)	
**Family history of hypertension** ^b^	25 (11.0)	8 (3.5)	0.00
**Smoking** ^b^	144 (63.2)	129 (56.6)	0.15
**Alcohol addiction status** ^b^	31 (13.6)	29 (12.7)	0.78
**BMI, (Kg/m^2^)** ^c^ <24^b^ ≥24^b^	22.6±3.6 151 (66.2) 74 (32.5)	20.9±3.3 192 (84.2) 36 (15.8)	0.00

a Data are presented as median (IQR),

b Data are presented as n (%),

c Data are presented as mean ± SD.

HTN, the hypertension group; Non-HTN, the non-hypertension group; BMI, body mass index.

Regarding HIV-related factors, the hypertensive group showed higher CD4^+^, CD8^+^ cell counts, CD4^+^/CD8^+^ ratio, longer time to HIV diagnosis, and duration of ART. In addition, more patients with undetectable viral loads (<100 copies/mL) were observed in the hypertensive group (**Table 2**).

**Table 2:** HIV-related factors of Patients in Both Groups.

HIV-related factors	HTN (n=228)	Non-HTN (n=228)	*P* Value
**HIV diagnosis, years** ^a^	9 (5,11)	5 (3,8)	0.00
1~4^b^	45 (19.7)	108 (47.4)	
5~9^b^	78 (34.2)	70 (30.7)	
≥10^b^	105 (46.1)	50 (21.9)	
**ART duration, years** ^a^	7 (4,10)	4 (2,7)	0.00
1~4^b^	63 (27.6)	124 (54.4)	
5~9^b^	85 (37.3)	66 (28.9)	
≥10^b^	80 (35.1)	38 (16.7)	
**Recent CD4^+^cell count (cells/µL)** ^a^	397.1 (277.1, 589.3)	291.0 (133.9, 519.7)	0.00
**Recent CD8^+^cell count (cells/µL)** ^a^	633.2 (442.6, 883.8)	553.8 (353.5, 824.9)	0.02
**Recent CD4^+^/CD8^+^ ratio** ^a^	0.7 (0.5, 1.0)	0.5 (0.3, 1.0)	0.00
**HIV viral load < 100 copies/mL** ^b^	170 (74.6)	141 (61.8)	0.00

a Data are presented as median (IQR),

b Data are presented as n (%).

HTN, the hypertension group; Non-HTN, the non-hypertension group; HIV, human immunodeficiency virus; ART, antiretroviral therapy.

**Figure 2:**
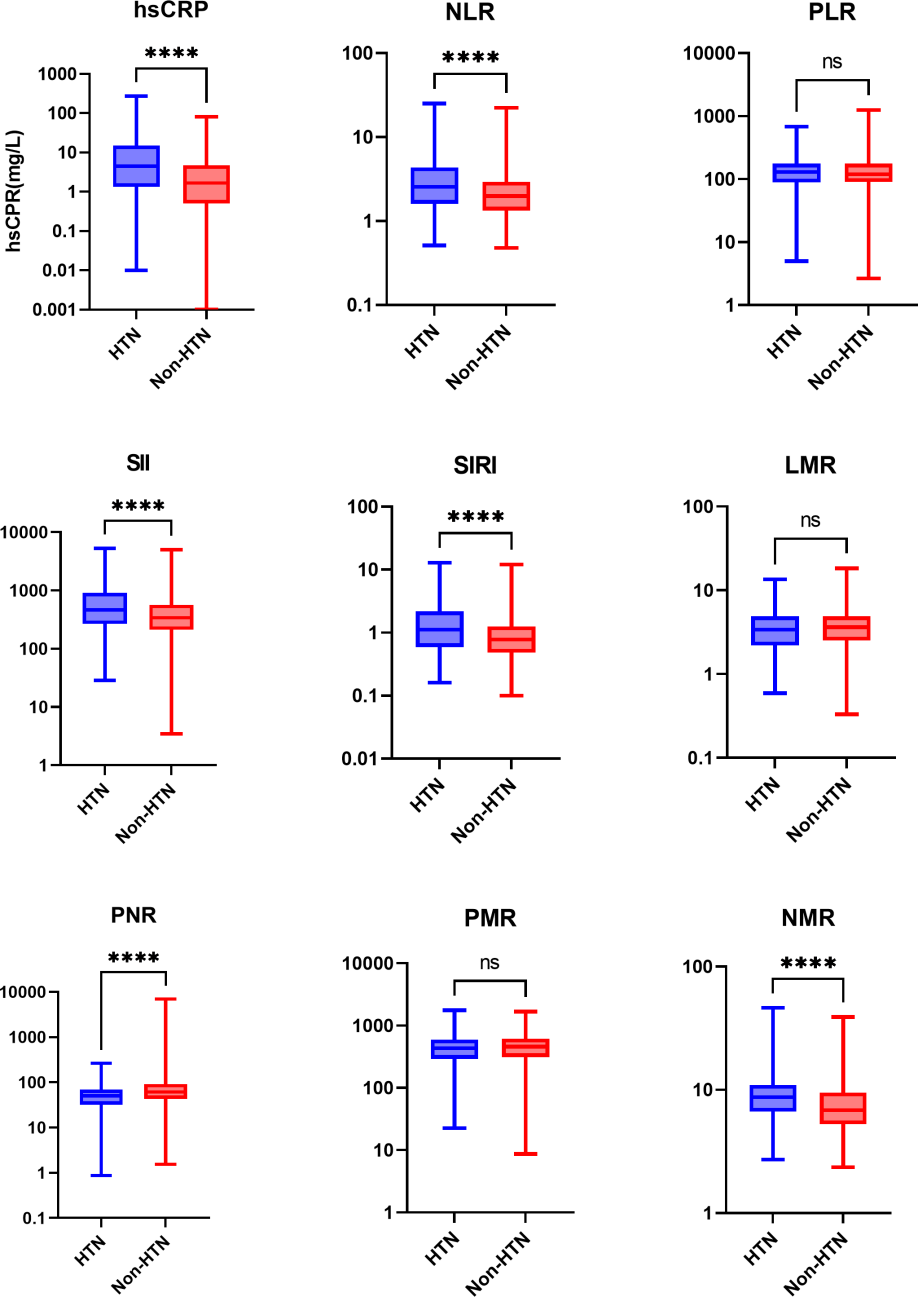
Boxplots comparing the inflammation marker levels of the two groups. Values are presented as median (IQR). log-transformed, ****P<0.0001, and ns, not significant. HTN, the hypertension group; Non-HTN, the non-hypertension group; hsCRP, high-sensitivity C-reactive protein; NLR, neutrophil-to-lymphocyte ratio; PLR, platelet-to-lymphocyte ratio; SII, systemic immune-inflammation index; SIRI, systemic inflammation response index; LMR, lymphocyte-to-monocyte ratio; PNR, platelet-to-neutrophil ratio; PMR, platelet-to-monocyte ratio; NMR, neutrophil-to-monocyte ratio.

Regarding the inflammation state, we compared the levels of inflammation markers in the two groups: hsCRP, NLR, SII, SIRI and NMR in the hypertension group were significantly higher than those in the non-hypertension group, and PNR was lower than that in the non-hypertension group. The PLR, LMR and PMR levels were no significant differences (**Table 3**). Among them, hsCRP, NLR, SII, SIRI, PNR, and NMR showed the most obvious differences (**Figure 2**).

**Table 3:** Inflammation markers of Patients in Both Groups.

Inflammation markers ^a^	HTN (n=228)	Non-HTN (n=228)	*P* Value
hsCRP	4.4 (1.3, 15.0)	1.7 (0.5, 4.7)	0.00
NLR	2.6 (1.6, 4.4)	2.0 (1.3, 2.9)	0.00
PLR	129.0 (88.9, 177.7)	120.2 (90.0, 177.3)	0.63
SII	469.3 (266.9, 909.5)	339.5 (211.9, 568.0)	0.00
SIRI	1.1 (0.6, 2.2)	0.8 (0.5, 1.3)	0.00
LMR	3.4 (2.2, 4.9)	3.6 (2.5, 4.9)	0.13
PNR	50.3 (32.0, 69.0)	60.9 (43.4, 92.0)	0.00
PMR	432.1 (292.4, 595.9)	457.7 (313.0, 612.2)	0.20
NMR	8.7 (6.7, 10.9)	6.8 (5.3, 9.5)	0.00

a Data are presented as median (IQR).

HTN, the hypertension group; Non-HTN, the non-hypertension group; hsCRP, high-sensitivity C-reactive protein; NLR, neutrophil-to-lymphocyte ratio; PLR, platelet‐to‐lymphocyte ratio; SII, systemic immune-inflammation index; SIRI, systemic inflammation response index; LMR, lymphocyte-to-monocyte ratio; PNR, platelet-to-neutrophil ratio; PMR, platelet-to-monocyte ratio; NMR, neutrophil-to-monocyte ratio.

### 3.2 Risk factors for hypertension

The result showed that higher hsCRP, SIRI, NMR, higher recent CD4^+^ cell counts and recent HIV-RNA of less than 100 Copies/mL were associated with hypertension. Patients with a BMI more than 24Kg/m^2^ (compared with BMI<24Kg/m^2^) had a 5-fold increased risk of developing hypertension. Compared with 1 to 4 years ART duration, ART duration of 5 to 9 years and over 10 years increased risk of hypertension 7-fold and 12-fold, respectively. However, recent CD8^+^ cell counts and CD4^+^/CD8^+^ ratio was negatively associated with risk of hypertension (**Table 4**).

**Table 4:** Factors associated with the risk of hypertension.

Factors	OR (95% CI)	*P* value
Demographic parameters		
BMI≥ 24, Kg/m^2^	4.9 (2.0,11.9)	0.00
**Inflammation markers**		
hsCRP	1.1 (1.1,1.2)	0.10
SIRI	1.4 (1.1,1.9)	0.01
NMR		
HIV-related factors		
ART duration, years		
1~ 4	1.0	
5~ 9	7.4 (2.5,22.4)	0.00
≥10	11.9 (3.2,43.9)	0.00
Recent CD4^+^cell count(cells/µL)	1.1 (1.1,1.2)	0.00
Recent CD8^+^cell count(cells/µL)	0.9 (0.8,0.9)	0.02
Recent CD4^+^/CD8^+^ ratio	0.1(0.1,0.6)	0.01
HIV viral load < 100 copies/mL	3.0 (1.1,8.4)	0.04

BMI, body mass index; hsCRP, high-sensitivity C-reactive protein; SIRI, systemic inflammation response index; NMR, neutrophil-to-monocyte ratio; HIV, human immunodeficiency virus; ART, antiretroviral therapy.

### 3.3 Correlation between inflammation markers and HIV-related factors

**Figure 3:**
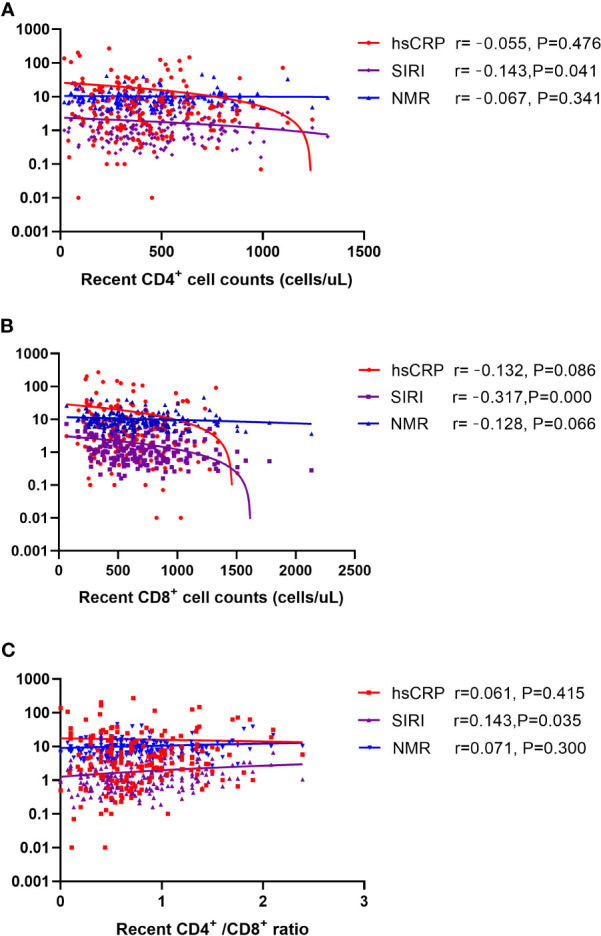
Scatter plot of the correlation between hsCRP, SIRI, and NMR with **(A)** recent CD4^+^ cell counts **(B)** recent CD8^+^ cell counts **(C)** CD4^+^/CD8^+^ ratio. Note: The ordinate is log transformed. Abbreviations: hsCRP, high-sensitivity C-reactive protein; SIRI, systemic inflammation response index; NMR, neutrophil-to-monocyte ratio.

Spearman’s correlation analysis was performed to explore the relationships between inflammation markers (hsCRP, SIRI, and NMR) and HIV-related parameters (recent CD4^+^ and CD8^+^ cell counts and their ratio). As shown in **Figure 3**, SIRI was negatively correlated with CD4^+^ cell counts and CD8^+^ cell counts, but positively correlated with CD4^+^/CD8^+^ ratio. Among them, the correlation between SIRI and CD8^+^ cell counts is the most obvious (**Figure 3**).

## 4 Discussion

The main findings of this study are as follows (1): an ART duration of 5 to 9 years or ≥10 years (compared with 1 to 4 years), CD4^+^ cell counts, HIV-RNA<100 copies/mL, hsCRP, SIRI, and NMR were positively associated with the prevalence of hypertension in PLWH, while CD8^+^ cell counts and CD4^+^/CD8^+^ ratio was negatively associated with the prevalence of hypertension in PLWH (2). high BMI was also associated with hypertension in PLWH. Among these factors, SIRI and NMR were associated with hypertension, independent of traditional risk factors. Previous studies have hypothesized that ethnic differences in the prevalence of hypertension may be related to dietary habits. However, Ruan et al.’ s research did not confirm this conjecture, they speculated that adaptation of different ethnic population to the local environment and dietary habits reduced the discrepancy in the prevalence of hypertension (25). Similar results were found in the PLWH population. Psychopathological factors caused by being single or poor marriage may indirectly lead to hypertension (26), but we found that in PLWH, marital status has no effect on the prevalence of hypertension. we considered that the mental damage caused by HIV is considerable, which weakens the impact of marriage on hypertension. In fact, HIV-specific factors combine with traditional risk factors to contribute to the high likelihood of PLWH developing hypertension (27). Several studies have demonstrated that inflammation biomarkers, such as interleukin (IL)-6 (28), IL-17, and tumor necrosis factor-α receptor 1(TNF-αR1) are associated with hypertension in PLWH (29). However, it is difficult to obtain the levels of these indicators clinically. We first demonstrate our results regarding easily measurable inflammation markers, such as hsCRP, SIRI, and NMR, which are associated with hypertensive risk in PLWH.

Inflammation is a defense mechanism against infection and trauma, which can stimulate the activation of innate and adaptive immune responses to fight pathogens and restore damaged tissues to their normal state. However, in some cases, chronic inflammation does not completely resolve and may persist across the lifespan (5). A prominent characteristic of HIV infection is the expression of proinflammatory cytokines, which regulate HIV replication and T cell apoptosis during the HIV lifecycle (30). Persistent low-level inflammation causes endothelial dysfunction and promotes the development of hypertension by activating inflammatory pathways and inducing oxidative stress (31). In PLWH, HIV replication is significantly inhibited under ART but does not completely disappear, with the residual virus replicating at low levels over long periods of time, which eventually causes long-term micro-inflammation. Impaired immune and activate inflammatory pathways induce oxidative stress, leading to endothelial dysfunction, which is a key factor in the pathogenesis of hypertension (5, 31). We found that hsCRP is a risk factor for hypertension in PLWH. hsCRP is one of the simplest and most direct indicators of inflammation in clinical practice, and achieves high sensitivity at low costs. In PLWH, elevated hsCRP may be due to HIV or other infections, and may resolve with successful treatment (32). Therefore, we can monitor the progress of low-level inflammation to predict the occurrence of hypertension, and implement early intervention to reduce effects.

Blood analysis is a simple and an easily accessible tool in which monocyte, neutrophil, and lymphocyte counts can be evaluated and used as clinical indicators. SIRI is a more comprehensive marker for chronic low-grade inflammation based on monocyte, neutrophil, and lymphocyte counts (33). NMR is the ratio of neutrophils to monocytes. Neutrophils and monocytes belong to the innate immune system, while lymphocytes are representative of the adaptive immune response, all of which participate in the body’s immune response. Lymphocytes are also the main target cells of the HIV virus. Neutrophils mediate immune responses by phagocytosing pathogenic microorganisms, releasing antimicrobial proteins *via* degranulation, and producing extracellular traps. By interacting with other innate and adaptive immune cells to attenuate the level of inflammation (such as natural killer cells and T cells), neutrophils can also secrete reactive oxygen species, promote endothelial dysfunction, and directly participate in the occurrence of hypertension (34). Moreover, neutrophil counts are independently associated with hypertension (35, 36). Monocytes participate in the body’s immune response by inducing phagocytosis of pathogens, secreting cytokines, and presenting antigens. For PLWH, HIV can reduce the number of monocytes and increase the proportion of intermediate monocytes (37–40). Even long-term regular ART cannot completely restore the disordered monocytes subsets. Intermediate monocytes, also known as inflammatory monocytes, have a strong pro-inflammatory effect and can injure the vascular endothelium (41, 42), which can secrete inflammation factors leading to persistent systemic inflammation (43). Activated lymphocytes can also induce inflammation and damage the vascular endothelium by secreting cytokines, thereby promoting the occurrence of hypertension (31, 44). In PLWH, persistently low levels of inflammation lead to increased neutrophil levels and decreased monocyte and lymphocyte levels, which translates into high SIRI and NMR values. Therefore, SIRI and NMR can serve as economical and convenient parameters for evaluating the inflammation status of PLWH, and can indicate their susceptibility to hypertension.

Regarding HIV-related factors, our study found that recent elevated CD4^+^ cell counts was associated with an increased risk of hypertension. Similar to our findings, Xu et al. reported that CD4^+^ cell counts exceeding 350 cells/µL were associated with higher risks for hypertension (45). In the general population, CD4^+^ cells are critical to the pathophysiology of hypertension and can produce immunosuppressive cytokines to suppress immune response activation (46). After HIV infection, CD4^+^ cell counts drop dramatically and then rise again when ART is initiated (27). Low CD4^+^ cell counts have been associated with higher risks for hypertension in PLWH (47–49), which disagrees with our findings. In fact, we found that low CD4^+^ cell counts occurred shortly after HIV infection. We inferred that after ART, even though the number of CD4^+^ cells increased, there was still a functional defect and insufficient ability to produce immunosuppressive factors such as low levels of transforming growth factor (TGF-β) and IL-10 (46). Interestingly, we found that CD8^+^ cell counts were negatively correlated with hypertension. CD8^+^ cells can protect against atherosclerosis by inducing plaque cell apoptosis and inflammation (50). A number of HIV antigens are recognized by antibodies which are correlated inversely with CD8^+^ T cell activation and positively with CD4^+^ cell counts (51). Early research showed that PLWH share some markers of aging with older noninfected patients, such as increased inflammation marker levels, D-dimers, CD4^+^/CD8^+^ inversion, and proportions of memory CD8 ^+^ T cells (52). The CD4^+^/CD8^+^ ratio might represent a marker that would remind clinicians to distinguish, among regular ART patients, those who require more attentive monitoring for possible early onset complications (52).

Compared with 1 to 4 years, ART for 5 to 9 years or over 10 years increased the risk of hypertension in PLWH. As mentioned earlier, hypertension is a typical age-related disease, long ART time represents a longer survival period of participant to a certain extent, with the prolongation of survival period, the probability of hypertension may be increase correspondingly. Furthermore, although ART is highly effective at inhibiting HIV replication, it is not curative, persistent low level HIV replication will result in micro-inflammation, inflammation is a clear risk factor for hypertension. Interestingly, Low levels of HIV-RNA (lower than 100 copies/mL) was positively associated with the prevalence of hypertensive in PLWH. We speculate that low level of HIV also produce inflammation, moreover, it may be related to time. In our study, most participants had undetectable HIV-RNA, the median time of HIV diagnosis and the median ART duration in the case group were longer than those in the control group. This means that inflammation exists for a longer time and has a higher degree of accumulation in the case group. In fact, a similar conclusion was reached in the Data Collection on Adverse Events of Anti-HIV Drugs (D: A:D) Study, which found that HIV-RNA>100,000 copies/mL was associated with a decreased risk of hypertension (47). HIV single-stranded RNA have been recognized by Toll-like Receptors 7, which then destroy T cells and release inflammatory cytokines (53). HIV-mediated transactivation of transcription protein activates Nuclear Factor-Kappa B (NF-kB) and upregulates the expressions of inflammation cytokines and chemokines (53). Immune activation persists in PLWH under ART, and markers of inflammation (i.e.IL-6) predict mortality in atherosclerotic cardiovascular disease (54). BMI, a traditional risk factor for hypertension, also plays a positive role in PLWH. Weight changes in PLWH after ART initiation is very common. Studies have found that high BMI is negatively associated with mortality and positively with cardiovascular events in PLWH (55, 56). Gender and age were independent risk factors associated with high BMI after 48 weeks of ART (57), but BMI was not associated with traditional biomarkers of inflammation, such as hsCRP, soluble tumor necrosis factor (sTNFR)-2, soluble CD14(sCD14), IL-6 (58).

We also analyzed the correlations between CD4^+^, CD8^+^, CD4^+^/CD8^+^ ratio, and inflammation markers. The results revealed that only SIRI correlated with CD4^+^ cell counts, CD8^+^ cell counts, and the CD4^+^/CD8^+^ ratio. SIRI combines neutrophils, monocytes, and lymphocytes to represent the balance between inflammation activators and regulators in the host. Composite indicators are more stable than single indicators, and high SIRI status reflects mediated by monocytes and neutrophils and a weak anti-inflammatory response mediated by lymphocyte (59). To the best of our knowledge, this is the first study to report the role of SIRI in hypertension in PLWH and the correlations between SIRI and T cells. Our findings may be useful for evaluating the immune status of a PLWH indirectly, preliminarily from indicators in blood. We also separately analyzed the correlation between BMI and inflammation markers in the case group, there is no correlation between BMI and inflammation factors (**Supplemental Figure 1**).

The study has some inevitable limitations. This was a single-center study with a small sample size, and multi center research is needed to further verify the validity of our conclusions. Microbial translocation, chronic inflammation, immune reconstitution, and HIV-related renal disease all influence the development and progression of hypertension. In addition, the use of INSTIs has been reported to affect hypertension (60), due to the limited number of patients using INSTIs, we have not found the effect of drugs on hypertension for the time being. Indeed, the effect of drugs on blood pressure may require longer observation. Due to the limitations of a retrospective study, we were unable to match all the traditional risk factors for hypertension. Although we tried to adjust for confounding factors, the results should be interpreted with caution in clinical practice. In addition, we cannot completely eliminate the influence of unknown opportunistic infections on the results. We tried to select homogeneous cases when manually matching the control group, and the selected control group cases all underwent regular ART. These steps were performed in an attempt to eliminate the influence of confounding factors.

While some limitations exist, we believe that our findings have important clinical significance. To our knowledge, inflammation markers in this study are based on routine blood tests, making all indicators easily available at early stages in hospitals and most communities, and provide a convenient and economical way to monitor inflammation levels. hsCRP, SIRI, and NMR were associated with an increased risk of hypertension in PLWH. Therefore, regular monitoring of inflammation markers is necessary in PLWH to distinguish and identify individuals at high-risk for hypertension. Alleviating inflammation may contribute to controlling or delaying the occurrence of hypertension in PLWH.

## Data availability statement

The original contributions presented in the study are included in the article/**Supplementary Material**. Further inquiries can be directed to the corresponding authors.

## Ethics statement

The studies involving human participants were reviewed and approved by the Ethics Committee of the Third People’s Hospital of Kunming. Written informed consent for participation was not required for this study in accordance with the national legislation and the institutional requirements.

## Author contributions

HO-Y and H-YF wrote the draft of manuscript and contributed equally. YL was responsible for data analysis, and interpretation. H-JL and Y-RD was responsible for the design and provided supervision. Z-YX, RG, J-YD, Y-CM participated in data collection and critically revised the manuscript. All authors contributed to the article and approved the submitted version.
