# Supplementary material for: Inflammation markers and the risk of hypertension in people living with HIV
Source: Front Immunol. 2023 Mar 21;14:1133640. doi: 10.3389/fimmu.2023.1133640 (PMC10071023; doi:10.3389/fimmu.2023.1133640)
Supplement: Supplementary file 1 [file DataSheet_1.pdf]

Supplementary Material

1 Supplementary Figures and Tables

1.1 Supplementary Tables

Supplementary Table1. Recent ART regimen of Patients in Both Groups

Characteristic	HTN(n=228)	Non-HTN(n=228)	*P* Value
**Number of ART classes** [a]			0.12
One	18(7.9)	10(4.4)	
Two	208(91.2)	212(93.0)	
Three	2(0.9)	6(2.6)	
**NRTIs** [a]			0.63
No	10(4.4)	8(3.5)	
Yes	218(95.6)	220(96.5)	
**NNRTIs** [a]			0.77
No	87(38.2)	90(39.5)	
Yes	141(61.8)	138(61.5)	
**PIs** [a]			0.92
No	164(71.9)	163(71.5)	
Yes	64(28.1)	65(28.5)	
**INSTIs** [a]			0.11
No	211(92.5)	201(88.2)	
Yes	17(7.5)	27(11.8)	
**Regimens** [a]			0.59
TDF+3TC+EFV	89(39.0)	97(42.5)	
TDF+3TC+LPV/r	23(10.1)	31(13.6)	
AZT+3TC+EFV	28(12.3)	21(9.2)	
AZT+3TC+LPV/r	13(5.7)	11(4.8)	
AZT+3TC+NVP	15(6.6)	10(4.4)	
ABC+3TC+LPV/r	12(5.3)	11(4.8)	
Other	48(21.1)	47(20.6)	

Notes: [a] Data are presented as n (%).

Abbreviations: HTN, the hypertension group; Non-HTN, the non-hypertension group; ART, antiretroviral treatment; NRTIs, nucleoside reverse transcriptase inhibitors; NNRTIs, nonnucleoside reverse transcriptase inhibitors; PIs, protease inhibitors; INSTIs, integrase transfer inhibitors; TDF, tenofovir disoproxil fumarate;3TC, lamivudine; EFV, efavirenz; LPV/r, lopinavir/ritonavir; AZT, zidovudine; ABC, abacavir; NVP, nevirapine.

1.2 Supplementary Figures

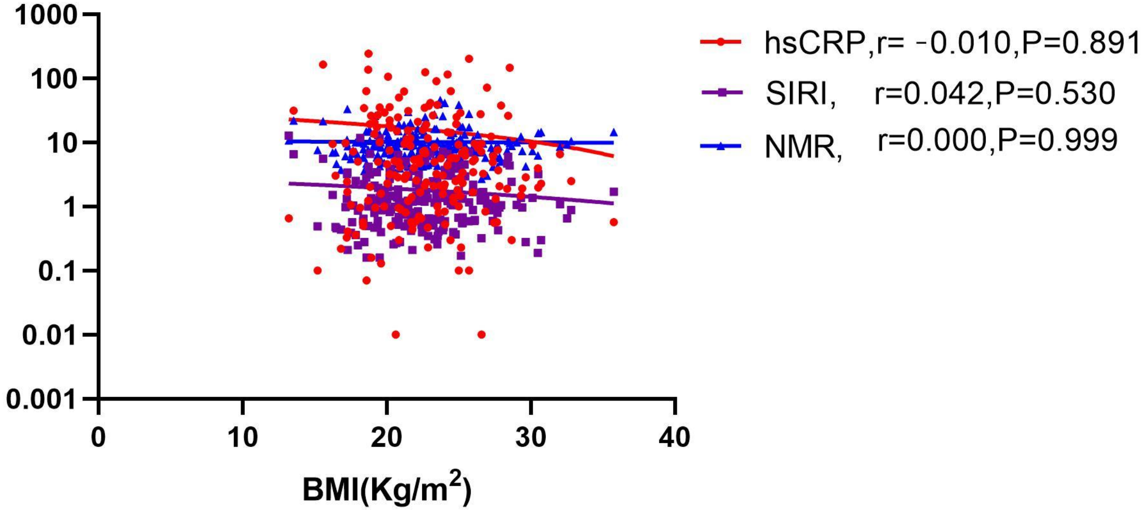

Supplementary Figure 1. Scatter plot of the correlation between hsCRP, SIRI, and NMR with BMI. Note: The ordinate is log transformed. Abbreviations: hsCRP, high-sensitivity C-reactive protein; SIRI, systemic inflammation response index; NMR, neutrophil-to-monocyte ratio; BMI, body mass index.
